# Does invasive *Chondrostoma nasus* shift the parasite community structure of endemic *Parachondrostoma toxostoma* in sympatric zones?

**DOI:** 10.1186/1756-3305-5-200

**Published:** 2012-09-11

**Authors:** Andrea Šimková, Petra Navrátilová, Martina Dávidová, Markéta Ondračková, Melthide Sinama, Rémi Chappaz, André Gilles, Caroline Costedoat

**Affiliations:** 1 Department of Botany and Zoology, Faculty of Science, Masaryk University, Kotlářská 2, 611 37, Brno, Czech Republic; 2 Aix-Marseille Université, IMBE, UMR CNRS 7263, Evolution Génome Environnement, Case 36, 3 Place Victor Hugo, 13331, Marseille Cedex 3, France

**Keywords:** Biological invasion, Endemic species, Cyprinid fish, Parasite communities, Monogenea, Hybrid zone

## Abstract

**Background:**

The composition of parasite communities in two cyprinid species in southern France – native and threatened *Parachondrostoma toxostoma* and introduced *Chondrostoma nasus* – was investigated. In sympatry, these two species form two hybrid zones in the Durance and Ardeche Rivers. Due to their different feeding preference and habitat positions in allopatry, we supposed a difference in parasite communities between fish species. We expected more similar parasite communities in sympatric zones associated with habitat overlap (facilitating the transmission of ectoparasites) and similar feeding (more generalist behaviour when compared to allopatry, facilitating the transmission of endoparasites) in both fish species. Finally, we investigated whether *P. toxostoma* x *C. nasus* hybrids are less parasitized then parental species.

**Methods:**

One allopatric population of each fish species plus two sympatric zones were sampled. Fish were identified using cytochrome *b* gene and 41 microsatellites loci and examined for all metazoan parasites.

**Results:**

A high Monogenea abundance was found in both allopatric and sympatric populations of *C. nasus*. Trematoda was the dominant group in parasite communities of *P. toxostoma* from the allopatric population. In contrast, the populations of *P. toxostoma* in sympatric zones were parasitized by *Dactylogyrus* species found in *C. nasus* populations, but their abundance in endemic species was low. Consequently, the similarity based on parasite presence/absence between the sympatric populations of *P. toxostoma* and *C. nasus* was high. Sympatric populations of *P. toxostoma* were more similar than allopatric and sympatric populations of this species. No difference in ectoparasite infection was found between *P. toxostoma* and hybrids, whilst *C. nasus* was more parasitized by Monogenea.

**Conclusions:**

The differences in endoparasites between *P. toxostoma* and *C. nasus* in allopatry are probably linked to different feeding or habitat conditions, but host-parasite evolutionary associations also play an important role in determining the presence of *Chondrostoma*-specific monogeneans. Our findings suggest that *Dactylogyrus* expanded with the source host *C. nasus* into introduced areas and that *P. toxostoma* became infected after contact with *C. nasus*. Although the genotype of *P. toxostoma* and recombinant genotypes of hybrids are susceptible to *Dactylogyrus* transmitted from *C. nasus*, the intensity of infection is low in these genotypes.

## Background

Biologists have long recognized that introduced species may have major effects on native communities. If an invading species occupies the same niche as a native species, strong interactions are likely to occur [1]. The introduction of species into novel areas and the formation of sympatric zones may affect the parasite diversity and distribution in native species. Introduced species may act as a competent host for a native parasite in which the infection is multiplied; then, the parasite “spills back” into the native host. This could result in an increase in the abundance of the native parasite and consequently in an increasing disease impact on native species [2]. By contrast, if an introduced species is resistant to a native parasite, it creates a dilution effect, lowering the parasite infection in the native host [3,4]. In addition, although the release from parasites and pathogens is considered to be a key factor explaining the successful expansion and survival of non-native species in the introduced regions (e.g. [5-7]), non-native parasite species may be introduced with their host, potentially threatening endangered and endemic local species [8]. Novel parasites introduced by the invader may remain host specific, or may be transmitted to native species. Especially in related host species, introduced parasites may represent a danger to native species, as they are expected to be adapted to the invader (e.g. [9]). In general, a change in patterns of parasitism may in turn affect host population dynamics.

After introduction events, the contact of native species with non-native relative species in sympatric areas often leads to the interspecific hybridization and formation of a hybrid zone. Hybridization becomes problematic especially for rare and endemic species [10]. Concerning the role of parasitism in hybrid zones, there have been a number of experimental and field studies investigating the resistance of different parental taxa and their hybrids to pathogens or parasites. Some of them using fish, amphibians or mammals as models have suggested that hybrids are less parasitized or more resistant to pathogen infection than their parental species [11-13]. However, other studies have indicated decreasing resistance as a result of hybridization processes [14-17]. It was proposed that hybrid susceptibility resulted from genomic incompatibilities between parental taxa [16]. Derothe *et al*. [17,18] postulated and experimentally confirmed the “parasite constraint” hypothesis, i.e. hybrid susceptibility is only applied to parasite species that have exerted enough constraints on their host to induce the selection of co-adapted genes among immune genes in the two parental genomes.

Many closely related cyprinid species living in sympatry tend to hybridize (e.g. [19-21]) and some of them form hybrid zones [22,23]. In cyprinids, several previous cases involving introduction of new species have led to the endangerment of native species. The situation with two particular cyprinid species – the native *Parachondrostoma toxostoma* and the introduced *Chondrostoma nasus* living in sympatry and forming two hybrid zones in the Durance River (including the Durance river plus the Buech river, which is a tributary) and the Ardeche River (South France, Rhone River drainage) – is such an example. *Parachondrostoma toxostoma* is a threatened, protected endemic cyprinid species in southern France. *Chondrostoma nasus* was introduced from Eastern Europe and colonized a part of the distribution range of *P. toxostoma*. The Durance hybrid zone of *P. toxostoma* and *C. nasus* has, until now, been studied more than the Ardeche hybrid zone. The Durance hybrid zone is of recent origin (around 100–150 years old) and represents a complex system with multiple effects including inter-species competition, bidirectional introgression, and environmental pressures [24]. The absence of a reproductive barrier between these two species found by Costedoat *et al*. [23] facilitates the hybridization between *P. toxostoma* and *C. nasus*.

Both species are morphologically and ecologically well differentiated in allopatry. Because of the different morphology of the mouth [25], *P. toxostoma* has a more generalist diet, feeding on algae and invertebrates, the latter being intermediate hosts for endoparasites, while *C. nasus* is a more specialized feeder, feeding mainly on benthic diatoms and algae in allopatric populations [26-29]. A difference in mouth morphology between allopatric and sympatric specimens and a convergence in mouth shape in the Durance hybrid zone were observed [30].

The aim of the study was to analyze the metazoan parasite communities of *P. toxostoma* and *C. nasus*. Due to different feeding preferences in allopatry, we supposed that allopatric *P. toxostoma* would be more parasitized by endoparasites than allopatric *C. nasus*. We also supposed differences in the composition of parasite communities between allopatric and sympatric populations. However, we expected the existence of more similar parasite fauna between *P. toxostoma* and *C. nasus* in sympatric areas where contact between the two species facilitates the transmission of ectoparasites, and we further expected that a shift to a larger generalist diet should facilitate the transmission of endoparasites. Finally, we investigated the parasite fauna of *P. toxostoma* x *C. nasus* hybrids and compared them with the parasite fauna of pure species.

## Methods

**Figure 1:**
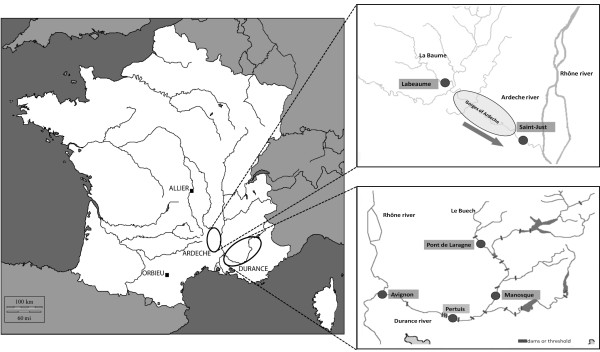
Positions of the localities investigated in southern France.

Four field studies (end of August 2008, June 2010, September 2010, and June 2011) were performed to collect *Parachondrostoma/Chondrostoma* specimens in southern France. One allopatric population of each species was sampled, *Parachondrostoma toxostoma* from the Orbieu River (Mediterranean Coastal river) and *C. nasus* from the Allier River (Loire drainage). Four localities on the Buech-Durance Rivers (Avignon, Manosque, Pertuis, Pont de Laragne) and two localities on the Ardeche River (Saint Just and Labeaume), where both fish species live in sympatry (two sympatric zones correspond to two hybrid zones in our study), were sampled (Figure 1, Table 1). The periods of investigation were selected to eliminate the effect of temporal variability (i.e. seasonality) on the composition of parasite communities and to exclude hot summers when fish collection and transport to the laboratory is difficult to perform. Fish were caught using electrofishing and transported live to the laboratory in barrels with the original oxygenated water. All fish were killed by severing the spinal cord shortly before dissection. The standard length (in millimeters) of each specimen was recorded. Complete dissection of fish was performed following Ergens & Lom [31]. Fish were examined for all metazoan parasites - ectoparasites (Monogenea, Crustacea, Mollusca and Hirudinea) and endoparasites (Trematoda, Cestoda, Acanthocephala and Nematoda). All parasites were removed, fixed as described in Rohlenová *et al*. [32], and determined to species/genus level using a light microscope (Olympus BX50) equipped with phase-contrast, differential interference contrast (DIC) and Digital Image Analysis (Olympus MicroImage^TM^ for Windows 95/98/NT 4.0 (Olympus Optical Co.)).

**Table 1:** **Locality description and molecular identification of *Parachondrostoma toxostoma *(PT), *Chondrostoma nasus *(CN), and hybrid (H) specimens**

** Localities**	**Type of population**	**N**	**Identification**
Allier	allopatric CN	11	11 CN
Orbieu	allopatric PT	11	11 PT
Le Buech (Pont de Laragne)	sympatric	41	17 PT, 19 CN, 5 H
Durance (Avignon)	sympatric	36	33 CN, 3 H
Durance (Manosque)	sympatric	68	49 PT, 9 CN, 10 H
Durance (Pertuis)	sympatric	22	20 PT, 1 CN, 1 H
Ardeche (Saint Just)	sympatric	30	11 PT, 18 CN, 1 H
La Baume (Labeaume)	sympatric	24	19 PT, 5 H

N represents the number of fish identified with molecular markers.

### Molecular identification

A fin sample from each fish specimen was preserved in 70% ethanol. All specimens were identified genetically using mitochondrial (5' part of the cytochrome *b* gene, as described in Costedoat *et al*. [33]) and nuclear markers (41 microsatellites distributed in five multiplex PCR kits, [34]). For microsatellites, amplification was performed for each kit in a total volume of 10 μL containing 1μL of total DNA extract using Qiagen Multiplex PCR Kit following the manufacturer’s protocol. Thermocycling was performed on a Mastercycler gradient (Eppendorf) with the following protocol: 95°C for 10 min, followed by 30 cycles (94°C for 1 min, 57°C for 1 min, 72°C for 1 min), and 60°C for 45 min. Visualization of the amplicons was conducted in an ABI 3130 Genetic Analyzer (Applied Biosystems). Allele sizes were scored against an internal GeneScan-500 LIZ standard and genotypes were obtained using GeneMapper® 3.7 [35].

### Data analyses

MicroChecker 2.2.3 [36] was used to check for null alleles, or scoring errors resulting from stuttering. NewHybrid 1.1 [37], a Bayesian clustering method, was used to assign specimens to genotypic classes: pure species (*C. nasus* and *P. toxostoma*) in allopatric and sympatric zones plus hybrids in sympatric zones. The Markov Chain Monte Carlo (MCMC) model was applied to compute all probabilities that one individual belongs to these different classes. The program was run five times with varying lengths of burn-in period and numbers of sweeps.

Adze 1.0 [38] was applied to estimate allelic richness and private allelic richness using the rarefaction method to compare populations with different sample sizes. The allelic richness of a population is the expected number of alleles in a sample of genes taken from a population. The private allelic richness is a convenient measure of how distinct a population is from other populations. Arlequin 3.1 [39] was used to estimate observed and expected heterozygosity by population. Genhet 2.3 [40] function implemented in R software (R development core team 2010) was used to calculate the standardized heterozygosity based on the mean expected heterozygosity and the standardized heterozygosity based on the mean observed heterozygosity. ANOVA with the Tukey post hoc test was used to test differences in heterozygosity between *P. toxostoma, C. nasus* and hybrids.

Similarity in parasite communities was calculated using the qualitative Jaccard index on the presence/absence matrix and the quantitative Morisita index on parasite abundance data [41]. General linear models (GLM) using the boostrap test with 1,000 permutations were applied to test differences in similarity among the following groups of populations (*C. nasus – C. nasus*; *P. toxostoma – P. toxostoma*; *C. nasus – P. toxostoma*) using (1) all populations and (2) only sympatric populations. Sample size and fish body length were included in GLM as covariates. Post hoc tests with Bonferroni correction were applied for multiple comparisons. In addition, GLM were applied to test the differences in similarity between “allopatric-sympatric” and “sympatric-sympatric” groups when the “sympatric-sympatric” group included the pairs of populations from both zones where *C. nasus* and *P. toxostoma* live in sympatry. This comparison was made for each fish species. The effect of host – i.e. *P. toxostoma*, *C. nasus* or hybrids – on parasite species richness or parasite abundance was tested using ANCOVA with fish body length and microsatellite heterozygosity as covariates. Parasite abundance and parasite species richness were log-transformed prior to GLM and ANCOVA. Statistical analyses were performed in Statistica 10 for Windows, StatSoft Inc and SPSS 20.0.0 (IBM Corporation, 2011).

## Results

### Molecular profiles of *Chondrostoma/Parachondrostoma* in localities

Using molecular determination (Table 1), an allopatric population of *C. nasus* was confirmed at Allier and an allopatric population of *P. toxostoma* was confirmed at Orbieu. Considering the localities where *P. toxostoma* and *C. nasus* live in sympatry, three different profiles based on molecular data were found: (1) localities with a high occurrence of *P. toxostoma* (i.e. Labeaume, Pertuis and Manosque), (2) localities with similar proportions of *P. toxostoma* and *C. nasus* (i.e. Pont de Laragne and Saint Just) and (3) a locality with a high occurrence of *C. nasus* (i.e. Avignon). The proportion of hybrids in localities with a sympatric occurrence of *C. nasus* and *P. toxostoma* was variable (i.e. from 3 to 20%). The highest proportion of hybrids was found at Manosque (Durance River) and Labeaume (Ardeche River), 16 and 20% respectively.

### Parasitism in sympatric and allopatric *Parachondrostoma/Chondrostoma* populations

**Figure 2:**
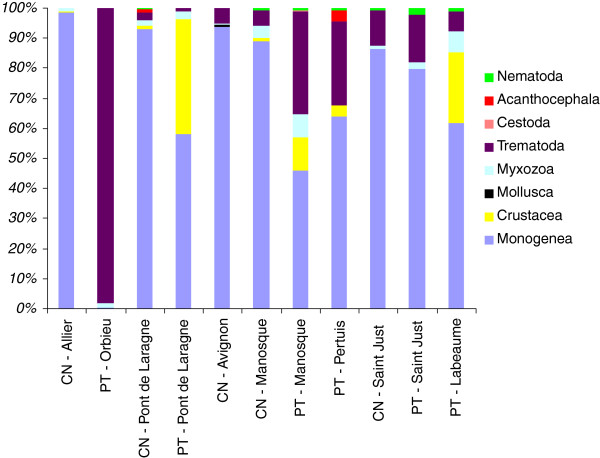
**Short title: Metazoan parasites in *C. nasus *and *P. toxostoma *populations.** Detail legend: Proportions of metazoan parasite groups in *C. nasus* and *P. toxostoma* populations. For sample size of each population, see Table 2.

A total of 11 fish populations were analyzed, two of which were considered to be allopatric and nine, sympatric (see Table 2 for the populations). Among parasite groups, Monogenea reached high proportions in the allopatric population of *C. nasus* and in all populations of both *P. toxostoma* and *C. nasus* in sympatric zones (Figure 2). However, a decrease in the proportion of Monogenea and an increase in the proportions of Crustacea and/or Trematoda were found in all *P. toxostoma* populations compared to *C. nasus* populations in sympatric zones. By contrast, Trematoda achieved the highest proportion in the allopatric population of *P. toxostoma*. The proportions of other parasite groups were low (Figure 2).

**Table 2:** **Microsatellite diversity and parasite diversity (Brillouin index) for *P. toxostoma *(PT) and *C. nasus *(CN) populations**

**Localities**	**Population**	**Sample size**	**Standard length**	**Allelic richness**	**Private allelic richness**	**Ho**	**He**	**Brillouin index of parasite diversity**
Orbieu	PT	11	176 ± 10.14	2.03 ± 0.17	0.15 ± 0.05	0.47 ± 0.27	0.49 ± 0.22	1.02 ± 0.22
Allier	CN	11	189.55 ± 28.44	3.03 ± 0.20	0.14 ± 0.04	0.61 ± 0.18	0.64 ± 0.17	0.87 ± 0.24
Pertuis	PT	20	128.65 ± 11.61	2.97 ± 0.30	0.26 ± 0.06	0.51 ± 0.31	0.53 ± 0.30	0.56 ± 0.39
	CN	1	100	-	-	-	-	-
Manosque	PT	49	113.61 ± 22.66	2.95 ± 0.29	0.26 ± 0.06	0.48 ± 0.30	0.50 ± 0.30	0.37 ± 0.36
	CN	9	130 ± 6.80	3.46 ± 0.23	0.23 ± 0.04	0.60 ± 0.26	0.66 ± 0.22	0.75 ± 0.46
Avignon	PT	-	-	-	-	-	-	-
	CN	33	203.41 ± 37.01	3.39 ± 0.23	0.16 ± 0.04	0.61 ± 0.21	0.65 ± 0.20	0.63 ± 0.37
Pont de Laragne	PT	17	117.24 ± 15.41	2.84 ± 0.29	0.23 ± 0.06	0.54 ± 0.30	0.55 ± 0.28	0.44 ± 0.41
	CN	19	155.47 ± 43.75	3.27 ± 0.22	0.15 ± 0.03	0.63 ± 0.22	0.64 ± 0.19	0.42 ± 0.39
LaBeaume	PT	19	175.89 ± 18.77	3.03 ± 0.29	0.25 ± 0.06	0.54 ± 0.30	0.52 ± 0.31	1.31 ± 0.23
	CN	-		-	-	-	-	
Saint Just	PT	11	108.18 ± 10.31	2.93 ± 0.30	0.27 ± 0.09	0.53 ± 0.31	0.55 ± 0.29	0.61 ± 0.47
	CN	18	113.22 ± 25.96	3.43 ± 0.23	0.19 ± 0.04	0.64 ± 0.24	0.65 ± 0.20	1.04 ± 0.37

Ho – observed heterozygosity, He – expected heterozygosity. Mean with standard deviation are shown.

There was a trend towards higher allelic richness and heterozygosity and lower private allelic richness in *C. nasus* populations compared to *P. toxostoma*. However, such a trend was not found for parasite diversity (Table 2). *Dactylogyrus* achieved the highest parasite abundance and prevalence in the allopatric population of *C. nasus* and all populations of *C. nasus* in sympatric zones (Table 3), but its abundance in the populations of *P. toxostoma* in sympatric zones was low (four populations) or reached a moderate value (Labeaume) (Table 4). *Dactylogyrus ergensi* was the most abundant species in parasite component communities of *C. nasus* from the allopatric population and three sympatric populations (Table 3). *Dactylogyrus dirigerus* and *Dactylogyrus* new sp. (this species is morphologically closely related to *D. ergensi* and *D. dirigerus*) were dominant in the parasite component community of *C. nasus* from Pont de Laragne (Table 3). *Dactylogyrus chondrostomi* was found in four populations of *C. nasus*, whilst this parasite species only rarely occurred in the *P. toxostoma* population at Manosque. In the allopatric population of *P. toxostoma*, only *Dactylogyrus vistulae* was found; this species achieved low abundance and prevalence (Table 4). Among Crustacea, *Ergasilus sieboldi* achieved high abundance and prevalence in two *P. toxostoma* populations (Table 4). Concerning endoparasites, *Diplostomum* spp. was the most abundant and prevalent in all populations of *P. toxostoma* and *C. nasus* in sympatric zones. The abundance of other endoparasite species in *P. toxostoma* and *C. nasus* populations in sympatric zones was low. In the allopatric population of *P. toxostoma*, four species of Trematoda were highly prevalent and abundant (Table 4).

**Table 3:** **Parasite abundance (A, mean ± standard deviation) and prevalence (P, in%) for each parasite species in *C. nasus *populations**

**Parasite species**	**Allier (11)**		**Pont de Laragne (19)**			**Avignon (33)**			**Manosque (9)**			**Saint Just (18)**		
	**A**	**P**	**A**	**P**	**H(5)**	**A**	**P**	**H(3)**	**A**	**P**	**H(10)**	**A**	**P**	**H(1)**
*Dactylogyrus chondrostomi*	2.27 ± 2.63	55	-	-		3.15 ± 5.29	70	*	1.22 ± 1.79	44		2.33 ± 4.47	56	
*Dactylogyrus dirigerus*	6.82 ± 9.61	73	7.42 ± 17.62	58	*	2 ± 2.41	64	*	1.11 ± 1.54	44	*	1.89 ± 2.47	56	*
*Dactylogyrus ergensi*	65.64 ± 77.79	100	-	-	*	44.91 ± 65.77	91	*	15.89 ± 12.62	89	*	9.5 ± 13.5	72	
*Dactylogyrus* new sp.	4.18 ± 6.01	64	19.16 ± 65.06	47	*	1.45 ± 2.43	52	*	3.33 ± 5.05	56	*	2.72 ± 2.52	67	*
*Dactylogyrus vistulae*	14.18 ± 15.33	100	0.26 ± 0.56	21	*	1.21 ± 1.62	52	*	0.11 ± 0.33	11	*	4.11 ± 3.94	89	*
*Gyrodactylus macrocornis*	-	-	0.26 ± 0.73	16	*	0.24 ± 0.56	18		2.78 ± 3.27	67	*	0.28 ± 0.75	17	
*Gyrodactylus pannonicus*	-	-	0.37 ± 1.12	11	*	-	-		-	-	*	-	-	
*Gyrodactylus paraminimus*	0.09 ± 0.29	9	-	-		0.09 ± 0.38	6		0.33 ± 0.71	22		0.61 ± 1.34	22	
*Paradiplozoon homoion*	-	-	-	-		-	-		-	-		0.06 ± 0.24	6	
*Paradiplozoon* new sp.	0.09 ± 0.29	9	-	-		0.09 ± 0.29	9	*	-	-		0.06 ± 0.24	6	
*Argulus foliaceus*	-	-	-	-		-	-		-	-	*	0.06 ± 0.24	6	
*Ergasilus sieboldi*	-	-	0.16 ± 0.50	11	*	-	-		0.33 ± 1	11	*	-	-	
*Lamproglena pulchella*	0.18 ± 0.39	18	0.16 ± 0.50	11		-	-		-	-		-	-	
*Anodonta* sp.	-	-	-	-		0.36 ± 1.19	12	*	-	-		-	-	
*Myxobolus* spp.	1.18 ± 2.21	27	0.63 ± 1.74	16	*	0.18 ± 0.73	9		1.11 ± 2.62	33	*	0.28 ± 0.46	28	
*Allocreadium markewitschi*	-	-	-	-		-	-		-	-		0.33 ± 1.41	6	
*Apharyngostrigea cornu*	-	-	-	-		-	-		-	-		0.06 ± 0.24	6	
*Clinostomum complanatum*	-	-	-	-		-	-		-	-		-	-	
*Diplostomum* spp.	-	-	0.74 ± 1.56	21		2.97 ± 6.87	39	*	1.44 ± 3.28	33		2.39 ± 3.68	56	*
Echinostomatidae fam. sp.	-	-	-	-		-	-		-	-		0.06 ± 0.24	6	
*Metorchis xanthosomus*	-	-	-	-		-	-		-	-		-	-	
*Paryphostomum radiatum*	-	-	-	-		-	-	*	-	-		0.11 ± 0.32	11	
Trematoda sp. metac.	-	-	-	-		-	-		-	-		-	-	
*Neogryporhynchus chleilancristrotus*	-	-	-	-		-	-		-	-		-	-	
*Pomphorhynchus terreticolis*	-	-	0.32 ± 0.82	16		-	-	*	-	-		-	-	
*Contracoecum* sp.	-	-	-	-		-	-		0.11 ± 0.33	11		-	-	
*Cucullanus* sp.	-	-	-	-		-	-		-	-		-	-	
*Philometra* sp.	-	-	0.05 ± 0.23	5		-	-		0.11 ± 0.33	11		-	-	
*Pseudocapillaria* sp.	-	-	-	-		-	-		-	-		-	-	
*Raphidascaris acus*	-	-	0.05 ± 0.23	5		-	-		-	-		0.17 ± 0.71	6	

The presence of parasite species in hybrids (H) is indicated by asterisks; sample size is included parentheses.

**Table 4:** **Parasite abundance (A, mean ± standard deviation) and prevalence (P, in%) for each parasite species in *P. toxostoma *populations**

**Parasite species**	**Orbieu (11)**		**Pont de Laragne (17)**			**Manosque (49)**			**Pertuis (20)**			**Saint Just (11)**			**Labeaume (19)**		
	**A**	**P**	**A**	**P**	**H(5)**	**A**	**P**	**H(10)**	**A**	**P**	**H(1)**	**A**	**P**	**H(1)**	**A**	**P**	**H(5)**
*Dactylogyrus chondrostomi*	-	-	-	-		0.04 ± 0.2	4		-	-		-	-		-	-	
*Dactylogyrus dirigerus*	-	-	0.18 ± 0.39	18	*	0.10 ± 0.47	6	*	0.4 ± 1.19	15		1.27 ± 1.74	55	*	5.79 ± 5.41	84	*
*Dactylogyrus ergensi*	-	-	0.65 ± 0.93	41	*	1 ± 1.79	39	*	0.45 ± 0.69	35		0.09 ± 0.3	9		4.63 ± 5.45	84	*
*Dactylogyrus* new sp.	-	-	0.35 ± 0.86	18	*	0.35 ± 0.81	22	*	0.25 ± 0.72	15		1.54 ± 1.44	64	*	6.11 ± 7.67	89	*
*Dactylogyrus vistulae*	0.09 ± 0.29	9	0.24 ± 0.56	18	*	0.29 ± 0.58	22	*	2.25 ± 2.69	75		2.27 ± 4	45	*	7 ± 5.65	89	*
*Gyrodactylus macrocornis*	0.09 ± 0.29	9	1.82 ± 6.26	29	*	0.37 ± 0.97	16	*	0.25 ± 0.72	15	*	0.27 ± 0.65	18		0.74 ± 1.33	32	*
*Gyrodactylus pannonicus*	-	-	5.71 ± 17	47	*	0.61 ± 2.75	16	*	0.3 ± 0.92	15		-	-		-	-	
*Gyrodactylus paraminimus*	-	-	0.06 ± 0.24	6		0.12 ± 0.33	12		-	-		-	-		0.05 ± 0.23	5	
*Paradiplozoon homoion*	-	-	-	-		-	-		0.45 ± 0.76	30		0.45 ± 0.82	27		-	-	
*Paradiplozoon* new sp.	-	-	-	-		-	-		-	-		0.55 ± 1.04	27		-	-	
*Argulus foliaceus*	-	-	-	-		-	-	*	-	-		-	-		0.32 ± 0.58	26	
*Ergasilus sieboldi*	0.09 ± 0.29	9	5.88 ± 17.33	41	*	0.63 ± 1.73	24	*	0.25 ± 0.72	15		-	-		8.95 ± 16.75	89	*
*Lamproglena pulchella*	-	-	-	-		0.06 ± 0.24	6		-	-		-	-		-	-	
*Anodonta* sp.	-	-	-	-		-	-		-	-		-	-		-	-	
*Myxobolus* spp.	0.82 ± 1.27	36	0.41 ± 1.23	18	*	0.49 ± 1.91	10	*	-	-		0.18 ± 0.6	9		2.68 ± 4.62	42	*
*Allocreadium markewitschi*	-	-	-	-		-	-		-	-		-	-		-	-	
*Apharyngostrigea cornu*	-	-	-	-		-	-		-	-		0.09 ± 0.3	9		0.05 ± 0.23	5	
*Clinostomum complanatum*	0.18 ± 0.39	18	-	-		-	-		-	-		-	-		0.63 ± 1.5	26	
*Diplostomum* spp.	13.36 ± 9.6	100	0.18 ± 0.53	12		2.12 ± 4.33	35		1.8 ± 2.24	55	*	1.18 ± 2.99	27	*	1.95 ± 2.61	58	*
Echinostomatidae fam. sp.	0.64 ± 2.01	9	-	-		-	-		-	-		-	-		-	-	
*Metorchis xanthosomus*	8.36 ± 9.72	82	-	-		0.02 ± 0.14	2		-	-		-	-		-	-	
*Paryphostomum radiatum*	29.36 ± 16.91	100	-	-		0.02 ± 0.14	2		-	-		-	-		-	-	
Trematoda sp. metac.	11.91 ± 10.30	91	-	-		-	-		0.1 ± 0.45	5		-	-		-	-	
*Neogryporhynchus chleilancristrotus*	-	-	-	-		0.02 ± 0.14	2		-	-		-	-		-	-	
*Pomphorhynchus terreticolis*	-	-	-	-		-	-		0.25 ± 0.64	15		-	-		-	-	
*Contracoecum* sp.	-	-	-	-		-	-		-	-		-	-		-	-	
*Cucullanus* sp.	-	-	-	-		-	-		0.05 ± 0.22	5		-	-		-	-	
*Philometra* sp.	-	-	-	-		0.04 ± 0.2	4		-	-		-	-		-	-	
*Pseudocapillaria* sp.	-	-	-	-		-	-		-	-		0.18 ± 0.6	9		0.47 ± 1.5	10	
*Raphidascaris acus*	-	-	-	-		-	-		-	-		-	-		-	-	

The presence of parasite species in hybrids (H) is indicated by asterisks; sample size is included in parentheses.

### Similarity in parasite communities in *Parachondrostoma/Chondrostoma* populations

**Figure 3:**
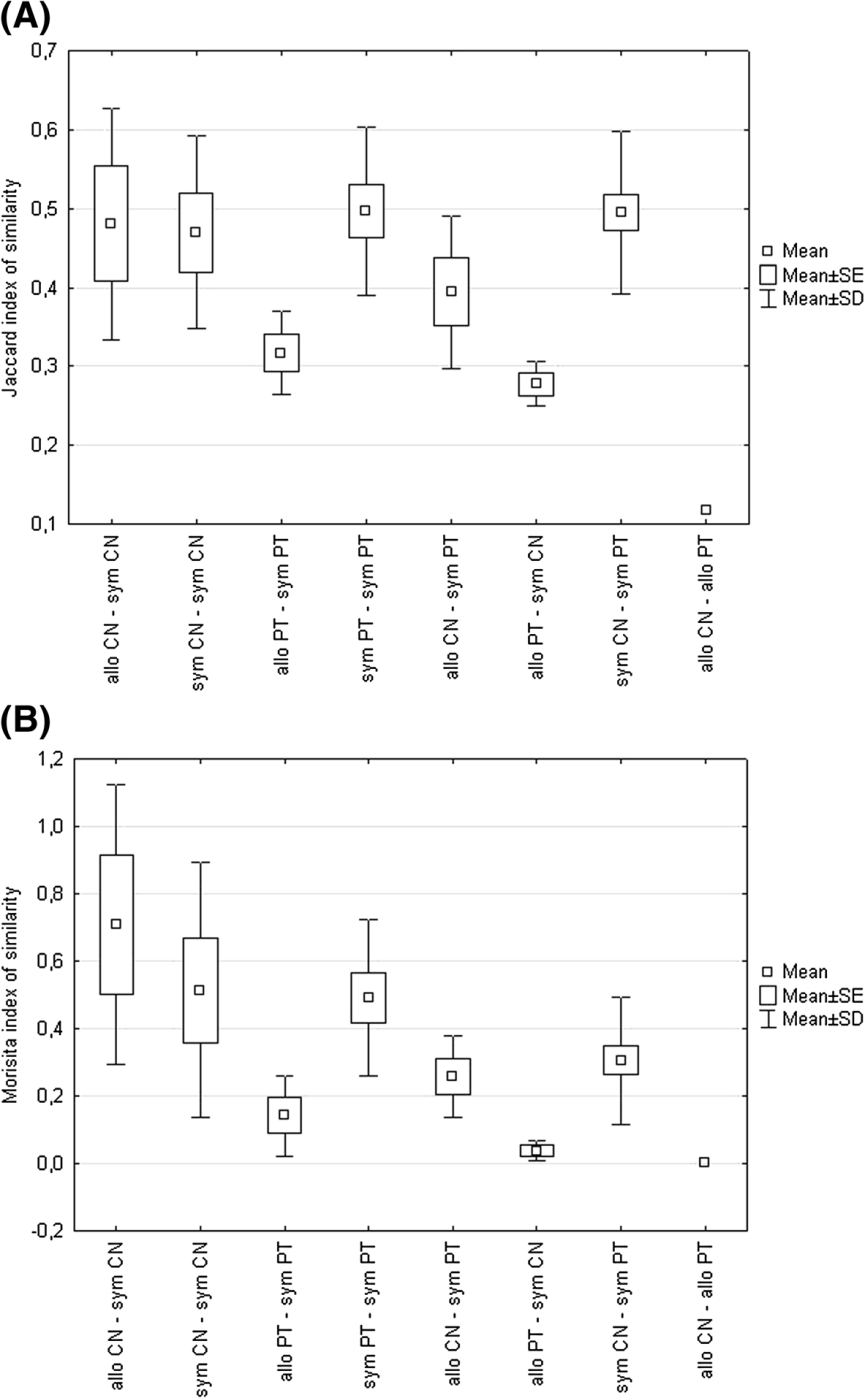
**Short title: Similarity in parasite communities based on parasite presence/absence data and parasite abundance.** Detail legend: Similarity in parasite communities based on parasite presence/absence data using Jaccard index (**A**), and parasite abundance using Morisita index (**B**) between different pairs of populations; allo – allopatric population; sym – sympatric population; CN – *Chondrostoma nasus*; PT – *Parachondrostoma toxostoma*.

The similarity of parasite communities based on parasite presence/absence data or parasite abundance for different pairs of populations is shown in Figure 3A-B. The lowest similarity in parasite communities was found between the allopatric population of *C. nasus* and the allopatric population of *P. toxostoma* using both parasite abundance and parasite presence data. When considering all populations, a significant difference in the similarity based on parasite abundance was found among the pairs of *C. nasus - C. nasus; P. toxostoma - P. toxostoma;* and *P. toxostoma – C. nasus* populations (whole model F_4,50_ = 3.60, p = 0.012, similarity F = 6.69, p = 0.003). Using the post hoc test, a significantly higher similarity was found only between *C. nasus - C. nasus* populations than between *P. toxostoma – C. nasus* populations (p = 0.003 after Bonferroni correction). No significant difference in similarity among the pairs of populations based on parasite presence data was found (p > 0.05). When comparing the similarity in parasite communities of *C. nasus* populations, no difference between allopatric–sympatric and sympatric–sympatric pairs of populations was found (p > 0.05). However, when comparing the similarity in parasite communities of *P. toxostoma* populations, a significantly higher similarity between sympatric-sympatric pairs of populations than between allopatric-sympatric pairs of populations was found using both parasite abundance (whole model F_3,11_ = 4.71, p = 0.024, similarity F = 12.26, p = 0.005) and parasite presence (whole model F_3,11_ = 3.68, p = 0.047, similarity F = 10.26, p = 0.008). Moreover, the similarity in parasite communities based on parasite presence/absence data between sympatric *P. toxostoma* and *C. nasus* populations was higher than that between allopatric and sympatric populations of *P. toxostoma* (whole model F_3,21_ = 6.18, p = 0.004, F = 14.90, p = 0.001). When considering only the populations from two sympatric zones, there was no significant difference in the similarity of parasite communities among *C. nasus - C. nasus*; *P. toxostoma - P. toxostoma*; or *P. toxostoma – C. nasus* pairs of populations based on parasite abundance or parasite presence data (p > 0.05). Host sample size and body length were insignificant in all GLM showed below.

### Parasitism in *P. toxostoma and C. nasus* hybrids

**Figure 4:**
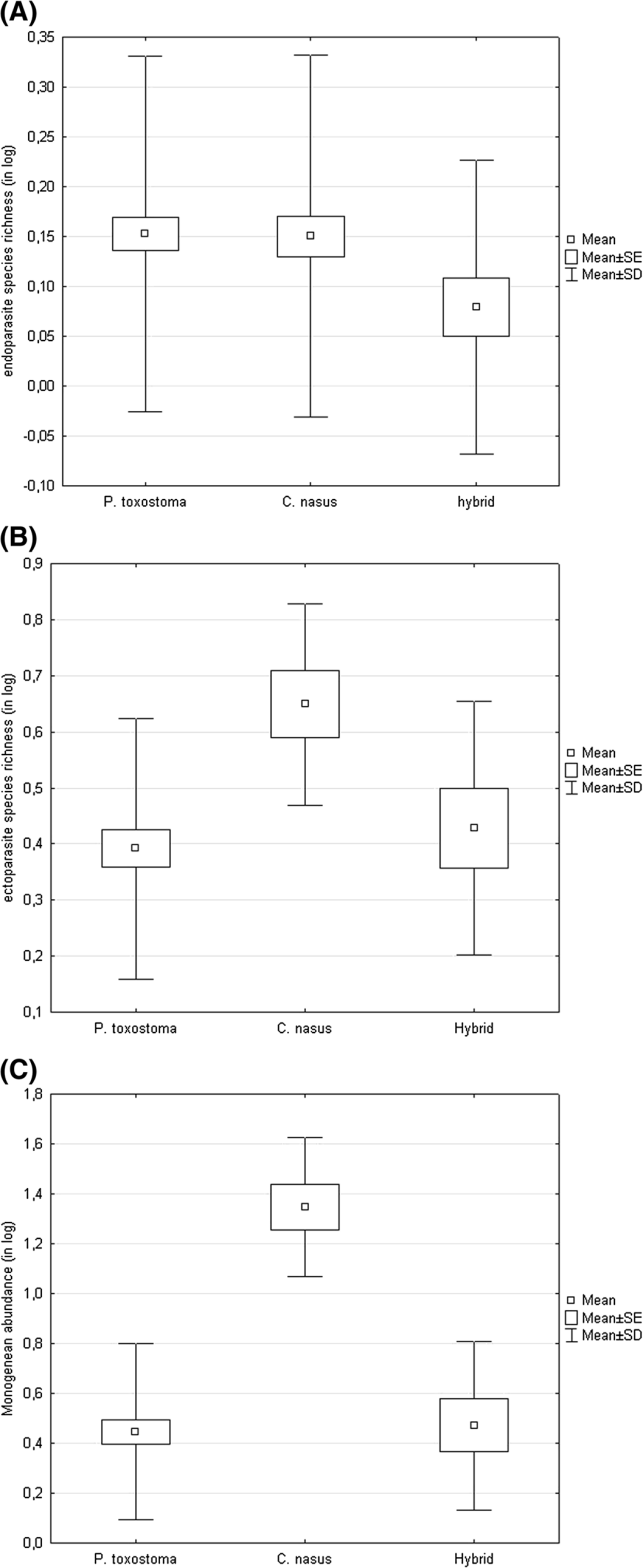
**Short title: Parasitism in *P. toxostoma*, *C. nasus *and hybrids.** Detail legend: Parasitism in *P. toxostoma*, *C. nasus* and hybrids measured by (**A**) endoparasite species richness using pooled data from sympatric zones and measured by (**B**) ectoparasite species richness and (**C**) monogenean abundance using data from Manosque (a locality in the Durance sympatric zone).

The molecular profiles of *Chondrostoma/Parachondrostoma* populations in hybrid zones (see Table 1) did not allow us to test simultaneously the effect of locality and effect of host on parasitism. Therefore, first, only the effect of host was tested using the dataset of all localities from sympatric zones. Microsatellite heterozygosity differed between *P. toxostoma*, *C. nasus* and hybrids (ANOVA, F_2, 220_ = 99.39, p < 0.001). The heterozygosity of hybrids was higher than that of both pure species and the heterozygosity of *C. nasus* was higher than that of *P. toxostoma* (p < 0.001). A significant effect of host on ectoparasite abundance was found (ANCOVA, F_4, 215_ total = 42.31, p < 0.001, heterozygosity F = 1.99, p = 0.159, body length F = 116.03, p < 0.001, host F = 5.01, p = 0.007). The Tukey post hoc test revealed significantly higher ectoparasite abundance in *C. nasus* when compared with *P. toxostoma* and hybrids (p < 0.001). The same result was found when using Monogenea or *Dactylogyrus* abundance. The prevalence of *Dactylogyrus chondrostomi* (a parasite species specific to *C. nasus*) in hybrids in the whole sample of sympatric zones was low (12%) and this species was found only in hybrids at Avignon. A significant effect of host on endoparasite abundance was also found (ANCOVA, F_4, 215_ total = 6.56, p < 0.001, heterozygosity F = 2.05, p = 0.153, body length F = 20.07, p < 0.001, host F = 3.25, p = 0.041). Using the Tukey post hoc test, no statistically significant difference was found between different pairs of *P. toxostoma*, *C. nasus* or hybrids. The same result was found when using endoparasite species richness. However, hybrids tended to harbour fewer endoparasite species and in lower abundance when compared to pure species (Figure 4A).

Because all measures of parasitism, i.e. parasite species richness and parasite abundance, were also affected by locality (ANCOVA, p < 0.001), we performed the next analysis to eliminate this effect. Manosque, among the localities situated in sympatric zones, was selected because the sample size in this locality was sufficient for statistical evaluation. A significant effect of host on ectoparasite species richness was found (ANCOVA, F_4,63_ total = 9.56, p < 0.001, heterozygosity F = 1.45, p = 0.23, body length F = 24.52, p < 0.001, host F = 3.33, p = 0.042). The Tukey post hoc test revealed a significantly higher ectoparasite species richness in *C. nasus* when compared with *P. toxostoma* (p = 0.001) and hybrids (p = 0.042) (Figure 4B). Unlike *P. toxostoma* and *C. nasus*, their hybrids harbour no endoparasite species. Considering parasite abundance, a significant effect of host on ectoparasite abundance was also found (ANCOVA, F_4,63_ total = 21.10, p < 0.001, heterozygosity F = 0.68, p = 0.413, body length F = 28.06, p < 0.001, host F = 17.90, p < 0.001). The Tukey post hoc test revealed significantly higher ectoparasite abundance in *C. nasus* when compared with *P. toxostoma* and hybrids (p = 0.001). The same result was found when using Monogenea (Figure 4C) or *Dactylogyrus* abundance. *Dactylogyrus chondrostomi* was not present in hybrids at Manosque.

## Discussion

In this study, we investigated metazoan parasite communities in two intergeneric cyprinid species, native and endemic *Parachondrostoma toxostoma* and invasive *Chondrostoma nasus*, sampled in allopatric and sympatric populations. Using mtDNA and microsatellites, we showed that the frequencies of *C. nasus* and *P. toxostoma* in the localities situated in sympatric zones varied from the more abundant *C. nasus* or more abundant *P. toxostoma* to an approximately balanced representation of the two species. This pattern of distribution of these two fish species in the sympatric zone of the Durance River was previously shown by Costedoat *et al*. [24]. We showed that the frequencies of *C. nasus* and *P. toxostoma* in the localities studied determine the composition of metazoan parasite communities, i.e. the localities with the highest frequencies of *C. nasus* were the localities with the highest proportion of Monogenea.

Specimens of *P. toxostoma* from the allopatric population were rarely infected by Monogenea. Instead, endoparasite species (i.e. mainly Trematoda) formed the dominant component of parasite communities. By contrast, *C. nasus* specimens from the allopatric population were not parasitized by endoparasite species and, in this case, Monogenea (especially *Dactylogyrus* species) formed the dominant component of parasite communities. The difference in endoparasite species richness between allopatric *P. toxostoma* and allopatric *C. nasus* could be explained by their different feeding preferences [26-29] (which are linked to different mouth morphology, following Corse *et al*. [25]) or by different abiotic and biotic factors of their habitats (i.e. the presence of invertebrates like mollusca, isopoda, ostracoda and copepoda, which serve as intermediate hosts for endoparasites). The difference in ectoparasite species richness observed between the allopatric population of *P. toxostoma* and the allopatric population of *C. nasus* could also indicate that allopatric *P. toxostoma* in southern France is rarely infected by monogenean species or is free of several monogenean species widely infecting *C. nasus*. However, to verify such a hypothesis, the further sampling of *P. toxostoma* from allopatric populations is needed. Our sampling was limited only to one allopatric population, taking into consideration the threatened and protected status of *P. toxostoma* in southern France. Up to now, the investigation of parasite fauna in the allopatric population of *P. toxostoma* has only been performed in the Viaur River (southwest France) by Loot *et al*. [42], who also found the low prevalence and abundance of *Dactylogyrus* and *Gyrodactylus* parasites. However, they found that *P. toxostoma* was infected only by two endoparasite species, which suggests that the endoparasite infection in our allopatric *P. toxostoma* is more likely the result of environmental conditions of the habitat.

In our study, we showed the high similarity between allopatric and sympatric populations of *C. nasus*, which may suggest that this species expands together with its original parasite fauna. The ectoparasites (especially *Dactylogyrus* species) maintain a similar intensity of infection in both allopatric and sympatric populations of *C. nasus*; thus, these parasites do not represent a factor limiting the survival and dispersal activity of their host species and their presence is probably the result of co-evolutionary host-parasite interactions, as shown for *Dactylogyrus* species and cyprinid fish by Šimková *et al*. [43]. On the other hand, low similarity in parasite communities between the allopatric and sympatric populations of *P. toxostoma* may indicate that *P. toxostoma* secondarily acquired the parasites (especially *Dactylogyrus*) after coming into contact with *C. nasus*. From this point of view, the changes in parasite communities in *P. toxostoma* linked to the invasion of *C. nasus* into areas originally inhabited solely by *P. toxostoma* could represent a danger for native endemic species. However, following the general trend of low *Dactylogyrus* abundance in *P. toxostoma* in relation to *C. nasus* observed in sympatric zones, it seems that *Dactylogyrus* infection probably has a genetic basis limiting the intensity of infection in endemic *P. toxostoma*. Among *Dactylogyrus* species, *Dactylogyrus chondrostomi* was found to be a species infecting *C. nasus* living in allopatric and sympatric populations with a high prevalence. This parasite species was absent in four populations and found only rarely in one population of *P. toxostoma* in sympatric zones, which indicates that *D. chondrostomi* is specific to *C. nasus*. It seems that higher parasite fitness (measured by parasite abundance) in *C. nasus* is associated with a system of co-adaptation genes.

On the basis of mtDNA and microsatellite data, different genotypes of *P. toxostoma* x *C. nasus* hybrids were identified. However, our results indicate that the proportion of hybrids and their genotypes across the two sympatric zones (the Durance River and the Ardeche River) are different. These results confirm the findings of a previous study in the Durance hybrid zone [24] and represent the first data obtained from the Ardeche River. The proportions of hybrids were unequal and sometimes low (ranging from 5 to 20%) depending on the considered localities. This fact together with the absence of *P. toxostoma* or *C. nasus* in some localities and the low frequencies of one of the *Chondrostoma* or *Parachondrostoma* species from other localities in the sympatric zones of the Durance and Ardeche Rivers did not allow us to test simultaneously the effect of locality and host on parasite species richness or abundance. Although parasite load was significantly influenced by the effect of locality, we showed the same pattern of parasite infection in hybrids at two different levels (1) using pooled data and (2) selecting one locality with the largest sample size within the Durance sympatric zone. Thus, *P. toxostoma x C. nasus* hybrids were less infected by ectoparasites (and especially by monogeneans of *Dactylogyrus* genus) than *C. nasus*. However, as no difference in ectoparasite abundance between hybrids and *P. toxostoma* was found, it seems that *Dactylogyrus* infection in *C. nasus* is more likely the result of co-evolutionary history between *C. nasus* and *Dactylogyrus* parasites, which limits the infection in both *P. toxostoma* and hybrids. Thus, both *P. toxostoma* and hybrids probably serve as additional hosts for *Dactylogyrus*. The susceptibility of *Salmo salar* x *Salmo trutta* hybrids to *Gyrodactylus salaris* (highly virulent) and *G. derjavini* (viviparous Monogenea) was experimentally tested and the intermediate pattern of hybrid susceptibility to that of the parents was shown [44]. They suggested that resistance was transferred through interspecific crosses as a dominant trait. However, our study seems to indicate that the low susceptibility of hybrids to *Dactylogyrus* infection is linked to the presence of *P. toxostoma* genes in recombinant genotypes. Metazoan parasite communities in another cyprinid hybrid system (*Alburnus alburnus* x *Rutilus rutilus* hybrids from Lake Micri Prespa, Northern Greece), were investigated by Dupont and Crivelli [45]. A higher susceptibility to metazoan parasite infection in hybrids compared to pure species was found for *Dactylogyrus* and *Diplozoon* species (Monogenea), *Bolbophorus confusus* (larval stages of Trematoda) and *Pomphorhynchus bosniacus* (Acanthocephala). This was explained by the spatial and trophic positions of the hybrids, which were intermediate between the two pure species. Concerning *P. toxostoma* x *C. nasus*, Corse *et al*. [30] concluded that pure and hybrid specimens in the hybrid zone exhibit more diverse feeding behaviour than in the allopatric zone consistent with generalist behaviour. *P. toxostoma* x *C. nasus* hybrids are not intermediate between pure species and hybrids have “super *P. toxostoma*” feeding behaviour i.e. they feed on fewer diatomes and more invertebrates than both pure species [46]. However, in our study, there was a trend towards lower endoparasite abundance in hybrids compared to both *P. toxostoma* and *C. nasus*, suggesting that there is no link between the frequencies of invertebrates (as the potential intermediate hosts) in the food and infection by endoparasite species in sympatric zones.

Concerning Monogenea, the strictly host-specific *Dactylogyrus* in the study by Dupont and Crivelli [44] achieved lower prevalence in *Alburnus alburnus* x *Rutilus rutilus* hybrids compared to pure species. A similar result, i.e. the effect of introgression rate on *Diplozoon* infection (Monogenea) in the hybrids of *Barbus barbus* and *B. meridionalis* from a hybrid zone in southern France, was found, in which parasite prevalence correlated with the percentage of *B. meridionalis* genes [47]. In our study, *Dactylogyrus chondrostomi*, a parasite specific to *C. nasus*, was absent in hybrids reported in five localities where both host fish species live in sympatry, and its presence was confirmed only in hybrids at Avignon, a single locality investigated in the sympatric zone on the Durance River, where 92% of collected specimens were determined as *C. nasus*. The high frequency of *C. nasus* seems to represent a more plausible explanation for the presence of *D. chondrostomi* in hybrids.

## Conclusions

We can conclude that the compositions of parasite communities in allopatric populations of *C. nasus* and *P. toxostoma* are very different, probably because of different feeding preferences or different abiotic and biotic characters of habitat (like the presence of intermediate hosts for endoparasites). However, they are also related to host-parasite co-evolutionary history, when the presence of a specific *Dactylogyrus* species on *C. nasus* seems to be the result of co-adaptation interactions. Our results suggest that *C. nasus* is a source of infection of *Dactylogyrus* parasites and has an impact on native and protected *P. toxostoma* with respect to their transmission. The genotype of *P. toxostoma* and recombinant genotypes of hybrids, even if they are susceptible to *Dactylogyrus*, have lower levels of *Dactylogyrus* infection than *C. nasus*.

## Competing interests

The authors declare that they have no competing interests.

## Authors' contributions

AS designed this study and drafted the manuscript. AŠ, MD, MO, RC, CC and AG participated on the field studies to acquire fish and parasite data. PN with the help of MD and MO determined the parasites. MS and CC carried out the molecular analyses and statistics on molecular data. AS analyzed the whole data. MD, MO, CC and AG have been involved in drafting the manuscript or revising it critically for important intellectual content. All authors read and approved the final version of manuscript.
